# Complex organisational factors influence multidisciplinary care for patients with hip fractures: a qualitative study of barriers and facilitators to service delivery

**DOI:** 10.1186/s12891-023-06164-9

**Published:** 2023-02-16

**Authors:** F Fox, S Drew, CL Gregson, R Patel, TJS Chesser, A Johansen, MK Javaid, XL Griffin, R Gooberman-Hill

**Affiliations:** 1 Bristol Medical School, University of Bristol, Bristol, UK; 2 Department of Trauma and Orthopaedics, North Bristol NHS Trust, Bristol, UK; 3 University Hospital of Wales and School of Medicine, Cardiff University, Cardiff, UK; 4 Nuffield Department of Orthopaedics, Rheumatology and Musculoskeletal Sciences, University of Oxford, Oxford, UK; 5 Bone and Joint Health, Barts and The London School of Medicine and Dentistry, Queen Mary University of London, London, UK; 6 Barts Health NHS Trust, London, UK; 7 National Institute for Health Research Bristol Biomedical Research Centre, University Hospitals Bristol and Weston NHS Foundation Trust and University of Bristol, Bristol, UK

**Keywords:** Hip fracture, Qualitative, Service delivery, Multidisciplinary care, Care pathway, Communication

## Abstract

**Background:**

Hip fractures are devastating injuries, with high health and social care costs. Despite national standards and guidelines, substantial variation persists in hospital delivery of hip fracture care and patient outcomes. This qualitative study aimed to identify organisational processes that can be targeted to reduce variation in service provision and improve patient care.

**Methods:**

Interviews were conducted with 40 staff delivering hip fracture care in four UK hospitals. Twenty-three anonymised British Orthopaedic Association reports addressing under-performing hip fracture services were analysed. Following Thematic Analysis of both data sources, themes were transposed onto domains both along and across the hip fracture care pathway.

**Results:**

Effective pre-operative care required early alert of patient admission and the availability of staff in emergency departments to undertake assessments, investigations and administer analgesia. Coordinated decision-making between medical and surgical teams regarding surgery was key, with strategies to ensure flexible but efficient trauma lists. Orthogeriatric services were central to effective service delivery, with collaborative working and supervision of junior doctors, specialist nurses and therapists. Information sharing via multidisciplinary meetings was facilitated by joined up information and technology systems. Service provision was improved by embedding hip fracture pathway documents in induction and training and ensuring their consistent use by the whole team. Hospital executive leadership was important in prioritising hip fracture care and advocating service improvement. Nominated specialty leads, who jointly owned the pathway and met regularly, actively steered services and regularly monitored performance, investigating lapses and consistently feeding back to the multidisciplinary team.

**Conclusion:**

Findings highlight the importance of representation from all teams and departments involved in the multidisciplinary care pathway, to deliver integrated hip fracture care. Complex, potentially modifiable, barriers and facilitators to care delivery were identified, informing recommendations to improve effective hip fracture care delivery, and assist hospital services when re-designing and implementing service improvements.

## Introduction

Approximately 70,000 people fracture their hip every year in the UK. Hip fractures are a devastating injury, responsible for a 20% persistent reduction in quality of life and high mortality rates [1, 2] with costs to healthcare services annually amounting to £1.2billion in the UK [3]. Hip fractures commonly occur in frail, older people with multimorbidity, complicating management and outcomes [4].Patients, who sustain hip fractures, require complex multidisciplinary care, to maximise survival and attenuate future fall and fracture risks [5].

The hip fracture care pathway involves multiple hospital departments and teams, spanning admission to discharge [5]. National guidelines have tried to standardise key components of this pathway [6–9]. Standards include admitting patients to an acute hip fracture ward; rapid optimisation of fitness for surgery; time-specific targets for surgery and first day post-operative mobilisation. Guidelines advocate continued, coordinated, orthogeriatric and multidisciplinary review, with the aim for patients to recover independence and return to pre-fracture residence [5, 6]. Adherence to these standards requires a multidisciplinary team who consistently communicate, collaborate [10, 11], understand the care pathway and are responsive to change.

Patients deserve the highest possible standard of care, whichever hospital they attend. However, despite improvements, care delivery and patient outcomes vary substantially between UK hospitals [12–14]. This qualitative study forms part of the NAME programme that examines sources of variation in fracture care delivery and the impact on patient outcomes [15].

Qualitative methods were used to investigate the organisational processes that help and hinder the implementation of hip fracture services. The aim was to identify potentially modifiable barriers and facilitators to care delivery, to inform recommendations to improve care delivery, assist service improvements, and ultimately reduce health inequities and improve patient outcomes.

## Methods

Data were collected and analysed via two sources: 1) one-to-one interviews with healthcare professionals delivering hip fracture care at four English hospitals, and (2) documentary analysis of anonymised British Orthopaedic Association (BOA) hospital reports addressing under-performing hip fracture services.

### Interviews with healthcare professionals

Four hospitals (three urban, one rural) were purposively selected to represent the delivery of different models of hip fracture care. Settings varied in terms of geography, number of hip fracture presentations per year, service configuration and audited hip fracture outcomes [15]. One hospital admitted a large number (> 75^th^ percentile), and three a moderate (between 25^th^-75^th^ percentile) number of hip fracture patients annually. To retain anonymity hospitals are identified by pseudonyms (see Table 1).

**Table 1:** Characteristics of Health Care Professional Interviewed

Professional Role	Number of professionals interviewed across each hospital				Time spent working in role
	**01 Springhill**	**02 Radford**	**03 Maplegrove**	**04 Newbridge**	
Consultant geriatrician / Orthogeriatrician	3	2	1	1	3—18 years
ED Consultant	1				5 years
Band 8 ANP Emergency Department		1			6 years
Anaesthetist	1	3	1	1	7—25 years
Orthopaedic surgeon	1	2	4		5—11 years
Orthogeriatric Advanced Nurse Specialist	1				14 years
Physiotherapist			1	1	3—5 years
Occupational Therapist	1	1	1	1	2—8 years
MSK Matron	1				7 years
Service Manager		1	1		5 years
Ward Manager		1			5 years
Senior Theatre Practitioner			1		8 years
ANP			2		13 – 17 years
Orthopaedic Registrar			1		5 years
FY2 Doctor			1		3.5 months
Discharge coordinator			1		19 years
Trauma coordinator				1	5 years
**Total**	**9**	**11**	**15**	**5**	

*Abbreviations*: *ANP* Advanced Nurse Practitioner, *ED* Emergency Department, *FY2* Foundation Year 2, *MSK* Musculoskeletal

### Sample and recruitment

At each hospital, local principal investigators identified a range of healthcare professionals delivering hip fracture care. The research team invited all to consider participation. To account for pressures experienced during the COVID-19 pandemic, criterion sampling was used to approach those most able to provide information about the study topics [16]. Selected staff included service managers, trauma and discharge coordinators and ward staff. The research team monitored and reviewed the data to ensure that the final sample size was sufficient to yield adequate ‘information power’ to address the research questions [17]. Of 75 individuals invited to participate, 40 (53%) were interviewed. The remaining 47% did not respond to the invitation and therefore their reasons for declining participation were unknown. as we did not have research ethics approval to collect this information. Non-responders came from a range of professional roles. It is likely possible that the pressures associated with the COVID-19 pandemic affected willingness and availability to participate in research interviews.

### One-to-one interviews

Before interviews, participants provided written informed consent. Two qualitative researchers (SD and FF) conducted interviews. Due to COVID-19 restrictions interviews were conducted by telephone (*n* = 21) or Microsoft Teams (*n* = 19) according to the individual participant’s preference. There was an even spread of interviews conducted by telephone and MS Teams at each site. Interviews, lasting 45 to 90 min, were steered by a topic guide that were co-developed in collaboration with four clinicians involved in hip fracture care and five patient representatives. Interviews explored the organisation and delivery of hip fracture services; barriers and facilitators to implementation of care; and multidisciplinary communication and cooperation.

### BOA reports

At the request of the study team, the BOA granted access to all reports of hospital-initiated peer-reviews conducted by the BOA for hospitals with under-performing hip fracture services. Twenty three anonymised reports were shared for 17 self-identifying under-performing hospitals between 2012 and 2019, and 6 hospitals involved in a quality improvement programme.

These reports appraised the hip fracture care pathway from admission to discharge via interviews with a range of staff, during two-day onsite visits. Reports identified areas of good practice and areas for improvement, with suggested action points. Three researchers (FF, SD and RGH) analysed the 23 anonymised reports.

### Analysis

Interviews were audio-recorded, transcribed by a professional transcription service (TTCUK) and anonymized by FF. Transcripts were analysed by FF in NVivo qualitative software, using an inductive approach to identify themes and subthemes [18]. To ensure rigour, two other qualitative researchers (SD and RGH) independently analysed 20% of transcripts, to agree a code list [18]. Data from the four hospitals were analysed as discrete datasets to enable comparison. Constant comparison was used to identify data relevant to each theme and to establish a coherent framework [18]. BOA reports were also analysed independently in NVIVO by three qualitative researchers (FF, SD, RGH), using the same inductive approach to identify themes.

Both the interview data and the BOA reports were coded to identify barriers and facilitators to service delivery. The themes from both sources were then amalgamated and transposed onto domains of Hip Fracture Care Pathway. Domains were grouped as: (a) along the care pathway: specific points of care during an acute admission, and (b) across the care pathway: factors affecting multidisciplinary patient care throughout all stages of admission. This iterative process was challenging as some themes fitted into multiple domains. The three researchers discussed and revised this framework until a coherent taxonomy of barriers and facilitators was achieved. A research stakeholder group, made up of 5 clinicians, and a Patient and Public Involvement (PPI) group, consisting of 5 orthopaedic patients met separately at several stages during the project. Initially the stakeholder group helped to identify areas for the interview topic guide. Following data analysis, each group met to discuss and prioritise the study findings.

## Results

### Characteristics of healthcare professionals

The 40 interviewees included staff from the emergency department; orthopaedics; anaesthetics; orthogeriatrics; Trust management; therapy and nursing teams. Between 5 and 15 participants took part from each hospital. Summarised participants’ characteristics are displayed in Table 1.

Findings are presented as key themes with emphasis on aspects of good practice that may reduce variation in care: 1) Along the care pathway: 1a) pre-operative assessment and care and (1b) post-operative care. 2) Across the pathway: (2a) communication and coordination and (2b) clinical governance and service improvement. Participant quotations, illustrating the themes are presented in Tables 2 and 3.

**Table 2:** Participant quotes: ALONG the care pathway

Theme	ALONG The care pathway	Pseudonym, PPT role & pseudonym of hospital
1. Pre-operative assessment and care Admission:	*“Our fascia iliaca block trolley is good. We were having problems where our equipment was all over the place and we were taking an awful lot of time to find the stuff to do the blocks to the extent that people just couldn't be bothered. We're incredibly busy in A&E and the amount of time people were finding trying to get the stuff was an issue. We found that we'd got a trolley that locks and has everything you would need in it. You bring the trolley to the patient to give the block … a number of nurse practitioners have competency packs now.”*	Lucy, ED Consultant Springhill
2: Wards	*“if you’ve got a separate ward, you would focus skills. People can find niches and can achieve greater things if you can give them a little bit more responsibility within an area like that and give them some ownership of it … I just think that a lot of these frail NOF patients would be better on a dedicated ward. It means that preoperatively you could make sure that all the appropriate investigations, preoperative things were done, skills of the staff could be enhanced, and post-operatively, if you’re managing relatively fewer patients, you can actually give a slightly more individualised care to the patients”*	George, Consultant Orthopaedic Surgeon, Maplegrove
3. Therapy and rehabilitation	*“now, we tend to do a lot more joint assessments and joint working and actually, I think it's better for the patient because it's all much more coordinated. You're getting two separate viewpoints but at the same time and then it's easier to come up with those discharge options and which route is going to be more suitable, or what to try next because it's much more coordinated. I think communication is improved a lot, again, over the past few years to make it work better”*	Chloe, Specialist Occupational Therapist, Maplegrove
4. Discharge	*“I like to get patients straight home. So that’s why I’m kind of involved with the therapy led, so we call it therapy led discharge now. So, we talk about their home circumstances, but I mean that’s the key thing to be discussing when the patient comes through the door is, ‘what’s home like? what is it we need to get you back there, and what services and what care have you got?’. So, it’s important to know their baseline so the therapists know what they need to aim for to go home”*	Joanne Trauma coordinator, Newbridge

**Table 3:** Participant quotes ACROSS the care pathway

Quote	ACROSS the care pathway	Pseudonym, PPT Role & hospital pseudonym
1. Communication and Coordination	*“I think it will come with somebody taking a lead role … with any team you want everybody to come together, but you need to have a person who clearly leads on this. So having a person in a clinical role who can lead on it along with somebody from management who can lead on it would be useful”*	Peter, consultant orthopaedic surgeon, Radford
2. Communication and Coordination	*PPT:” So, I don’t actually know what the targets are, well I was never told them or I was never explained them or anything. I only knew this because I saw one of my colleagues doing an audit, the national hip fracture audit or putting them on the national database, and I was like, ‘oh what's that you're doing’? And then she said, ‘oh yes, we have to operate on them within 48 h if we can, and that’s the national target’. So, I don’t think people are aware unless there's some kind of hearsay or they figure it out. But I think that is a good thing to know if possible …* *IV: why would it be particularly helpful for you to know about the expected targets?* PPT: “*So, obviously we’re only there for four months and you could spend a month and a half doing it wrong before you do it right and then you’ve only got two and a half months left trying to do it right. Or for example even as a junior it’s important for us to understand why it’s, from an education point of view, why it’s important to operate on a patient within 48 h, are there risks to not operating on them, what are the complications. Just as an education stand, is it a national target because of money or is it a national target because of patient care or is it a national target because of bed flow in the hospitals like, is there a reason behind this? So in that sense its quite important as well”*	Alice, F2 Doctor Maplegrove
3. Service improvement and clinical governance	*“a lot of the drive comes from the hip fracture pathway. That you’re on a bit more of a schedule. Whereas if you haven’t got a pathway, everything’s a bit wishy-washy. But I think the hip fracture pathway does motivate people. Because there is a constant pressure. Because it’s, ‘okay, it’s seven days after their operation, what’s happening? Why aren’t we progressing’? Because we’re an MDT team, you’re not left alone, there’s a constant drive from every member or profession of the MDT to progress that patient … So I think everyone has a bit of a collective drive, but I think that being on that ward and on the pathway is a mega drive. Because it’s constantly evaluated”*	Jane, Occupational Therapist, Springhill
4. Service improvement and clinical governance	*“we are always looking at our figures, we are always downloading the NHFD data to see, you know, are there any trends … and then between us we will look at it and go, ‘ooh you know we are getting a few delays here due to DOACs [direct oral anticoagulants]’, that kind of thing, so then we will just remind the team of the DOAC guidelines and so we are very proactive instead of reactive and you know she [HF lead] is always looking at that because we’ve had like letters in the past from the NHFG saying that we’re really, really good and all that kind of thing, I think because she likes that recognition – as do we all – it keeps us working so that we don’t let it all lapse***”**	David, Orthogeriatric Advanced Nurse Specialist, Springhill

### Along the hip fracture care pathway

#### Pre-operative assessment and care

##### Admission

Protocols to notify orthopaedic, orthogeriatric, anaesthetic and ward staff about the admission of hip fracture patients were advocated by participants. This early cross-disciplinary communication was crucial to ensure timely ward transfer, and to manage bed space and patient flow. In some hospitals Advanced Nurse Practitioners (ANPs) proactively screened Emergency Departments (ED) for hip fracture patients, conducted assessments, interpreted radiographs, administered pain control and ensured rapid ward transfers. In other hospitals lack of clarity over which team should take responsibility for admission caused delays. The process of ED admission was enhanced by consistent use of hip fracture pathway documents, which travelled with patients. These protocols for patient assessment, investigation, optimisation and pain management, enabled a variety of staff members to co-manage the admission, ensuring patients were fast tracked to the ward. The importance of reducing the number of potentially painful bed moves between ED, radiography and wards was highlighted.

Differences emerged between hospitals which standardised the provision of, and training for, fascia iliaca (FI) nerve blocks in the ED and those where this was not routine. In the absence of protocols, some staff identified uncertainty about patient suitability for nerve blocks and some hospitals lacked the ED staff trained to administer them. One hospital developed a designated trolley with all necessary equipment for delivering FI nerve blocks, trained a range of staff to deliver them and ensured reliable access to equipment (Table 2, Quote 1).

##### Pre-operative care and surgery

Shared responsibility, between orthogeriatric and orthopaedic teams, for hip fracture patients was crucial to deliver an effective and integrated care pathway. Adherence to established and regularly updated hip fracture protocols for patient assessment, investigation and optimisation ensured good preoperative care. This required coordination between medical, anaesthetic and orthopaedic surgical teams and synchronised multidisciplinary team (MDT) decision-making regarding surgery, gaining consent and decisions regarding cancellation.

Having a nominated anaesthetic lead for trauma generally, or hip fracture specifically, was advocated. Their engagement within the MDT included attendance at trauma meetings, although varied working hours was sometimes a barrier. Early anaesthetic review following admission facilitated effective pre-operative optimisation of patients. Inconsistent standards of care were attributed to variation in anaesthetic techniques, training and experience with hip fracture patients. The presence of hip fracture protocols and consultant supervision of junior anaesthetists went some way towards mitigating these issues.

Participants emphasised the importance of efficient trauma lists. Interview participants at one site recommended that if a seven-day trauma list could not be achieved, an ‘extra operating list on a Friday’ could be implemented to clear cases before weekend admissions. The ‘golden patient’ system was a term used by advocated for by interview participants at several sites. It indicates a patient, identified and prepared the day before, who is reviewed by an anaesthetist the previous day and able to go to theatre first thing in the morning (e.g. at 08.00). At one hospital the early publication of trauma lists (prior to the morning trauma meeting) helped meet time-to-theatre targets. A live electronic feed, from theatre to the ward, visible on a screen, improved the efficient flow of patients into surgery. To support trauma surgery provision, flexibility to use vacant elective theatres, or cancel elective activity was endorsed. The availability of surgeons with expertise in hip fracture surgery was crucial, as well as their active role in patient management both pre- and post-surgery.

##### Post-operative care

The provision of an appropriate staffed orthogeriatric service, with expertise in managing frail, older patients was crucial to ensure holistic care for hip fracture patients from admission to discharge. Participants underscored the need for orthogeriatricians to supervise and work collaboratively with ANPs, therapists and junior doctors (medical and surgical) to provide care, via MDT meetings, board rounds and huddles. The capacity of services was impeded by difficulty recruiting orthogeriatricians, poor job planning with insufficient time allocated for orthogeriatric responsibilities, and lack of orthogeriatric cover at weekends or periods of leave. Focused advertising of orthogeriatric posts through relevant specialist interest groups within the British Geriatric Society was advocated, along with clear job plans and adequate timetabling for this service domain.

##### Wards

Grouping patients together in designated hip fracture wards was proposed as a solution to facilitate the specialist care that these complex patients require (Table 2, Quote 2). Alternatively, ringfencing beds on trauma and orthopaedic wards was suggested, to ensure space for hip fracture patients and avoid the inefficiency of outliers. Findings identified the need for reliable IT and communication systems to monitor and manage outliers. Specific nursing expertise was highlighted to deliver effective hip fracture care, with key skills including: the ability to mobilise patients and awareness of ‘weight-bearing as default’ post-operatively, unless specified otherwise by surgeons; routine use of pressure-relieving mattresses and two-hourly positional changes. It was suggested that Health Care Assistants (HCAs) support improved nutrition through feeding patients and that specific staff could support patient wellbeing and dementia friendly wards. The importance of specialist training for all staff was highlighted, this included upskilling nurses in relevant aspects of hip fracture care and the induction, supervision and mentoring of rotating junior doctors.

##### Therapy and rehabilitation

Appropriately resourced therapy staffing, particularly out of hours and at weekends, was crucial for continuity of patient care. Where weekend therapy provision was lacking, the impact on patient rehabilitation was considerable, although this could be somewhat mitigated by nursing staff routinely mobilising hip fracture patients. Participants explained that therapy provision could be facilitated by a system to prioritise patients who were first-day post-operation. Services were most effective when physiotherapists and occupational therapists routinely undertook joint assessments and maintained shared plans for patient rehabilitation and discharge (Table 2, Quote 3). The progression of patient mobility, beyond the ability to stand, was improved by formal strength and balance training, facilitated by protocols.

##### Discharge

Participants thought that discharge planning was most efficiently managed by a discharge coordinator and that meetings should routinely involve a social worker, physio- and/or occupational therapist, ward staff and an orthogeriatrician. Agreeing a discharge destination as early as possible was key, facilitated by communication with, and early information gathering from, a patient’s family. Clear discharge pathways systems were advocated, ideally with a ‘home first’ approach (rather then transfer to another rehabilitation facility). Daily reviews of readiness for discharge were important and were successfully managed between the hospital and community settings via shared patient information systems. Separate IT systems impeded information transfer. Clear documentation of mobility progression, with a ‘therapy-led’ approach was identified as helpful for discharge planning (Table 2, Quote 4).

Delays to discharge of medically fit patients were caused by challenges to: organising packages of care, finding appropriate nursing or care home placements, arranging home adjustments, and lack of transport. It was highlighted that bone medication and falls prevention plans should be in place before discharge. However, the provision of a falls and fracture liaison services within hospitals varied. Services relied on consistent staff training to undertake falls assessments, which could be completed by ACPs and physiotherapists.

### Across the hip fracture care pathway

#### Communication and coordination

All the study sites used a Hip Fracture Pathway, although the development and consistent use of processes and documentation varied. The findings indicate that the delivery of an integrated hip fracture pathway required effective MDT interaction at ward, organisational and managerial levels. Senior interactions between orthopaedic, orthogeriatric, anaesthetic and nursing teams at regular hip fracture meetings provided opportunities to communicate, review standards and plan service improvements (Table 3, Quote 1). Such joint leadership offset traditional siloed working. Joint training involving multiple staff groups helped to improve working relationships. It was highlighted that therapy teams often felt excluded at organisational and managerial levels and that therapy representation could promote more collaborative and coordinated post-operative care.

Comprehensive hip fracture pathway documentation, co-designed collaboratively between specialist leads, needed to be consistently used by clinical staff. For this to happen, the documents had to be a core part of the induction and training for junior doctors and rotating staff, so the entire MDT were working towards the same targets and guidelines (Table 3, Quote 2).

Achieving these goals at a daily ward level was augmented by efficient patient information systems, for example: ward boards with key patient information, technical bed and discharge management tools, and live trauma boards. A variety of communication modes such as WhatsApp, email and mobile phones (rather than pagers) supported MDT information sharing and collective working. Physical proximity of MDT members, through shared office space, regular ‘huddles’ and, or ward-based meetings was an important factor in coordinating care. Day-to-day coordination of the hip fracture service was delivered via core staff, such as specialist nurses and trauma coordinators. It was suggested that hip fracture patients should be under the active joint care of orthopaedic surgeons and orthogeriatricians, cooperating from admission to discharge, to provide seamless, high-quality care.

Inadequate and inconsistent staffing provision created considerable issues for service delivery, especially at weekends and out-of-hours. Participants suggested that this could be addressed through access to physicians to address medical issues; a robust handover system to weekend and on-call staff; clear prioritisation of patients when therapist capacity was inadequate, and nursing staff skilled to mobilise patients at weekends.

#### Service improvement and clinical governance

Participants indicated the need for designated hip fracture leaders at hospital board executive level, who were visible and engaged in bidirectional communication with clinical staff. Along with clinical leads these individuals should be responsible for co-developing clinical governance processes for hip fractures and promoting services as a designated hospital priority. The allocation of funding for hip fracture services should be resourced to reflect the intensive nursing that this physically and mentally frail patient group requires.

Service improvement was most effectively driven by collaboration between hip fracture leads from orthopaedic, orthogeriatric, anaesthetic and nursing divisions. This required adequate time allocation within job plans, plus management training and managerial support to drive change. It was agreed that this degree of commitment facilitated a clear leadership and governance structure and responsibility. Participants believed that this approach would facilitate development of shared priorities and goals, joint protocols, audit priorities and quality improvement plans agreed across the care pathway. The embedded use of hip fracture pathway documentation and regular performance monitoring motivated MDT members to constantly reflect on each patient’s progress (Table 3, Quote 3).

The importance of having designated individuals responsible for consistent entry of national clinical audit data into the National Hip Fracture Database (NHFD) was highlighted. NHFD key performance indicators, process and outcomes should all be constantly monitored, and regularly fed back to the MDT (Table 3, Quote 4). Participants suggested an investigation should be automatically triggered when targets were not met. Having protected time to attend mortality reviews was important, with easy access to medical notes for review. Proactive attempts to understand reasons for increased mortality, including the stage in the care pathway at which patients deteriorated, was important, with recurring themes identified to guide improvements, fed back to the MDT.

## Discussion

These findings indicate that active joint care, where hip fracture patients are admitted to a specialised orthogeriatric ward under the combined care of orthogeriatric and orthopaedic teams [5, 9, 19], is likely to ensure seamless, high-quality care and improve post-operative mortality [20]. This is in keeping with strong evidence that co-management, where medical (geriatrician) and surgical (orthopedic) teams take equal responsibility for care [21], can lead to reduced length of hospital stay and improved outcomes for patients [4, 20].

The current findings support NICE guidance and research evidence, that coordinated multidisciplinary interaction is necessary to achieve acceptable standards for effective hip fracture care [6, 7, 9, 22, 23]. Our study identifies that sustained commitment from clinical leads, with ongoing support from senior/executive hospital Trust leaders, creates the foundation of a collectively-owned effective hip fracture service. The involvement of clinical leads has been highlighted elsewhere [5] (although the significance of executive level support is less well documented. Evidence suggests that in the absence of a collective hip fracture pathway, the care journey is divided into service delivery units resulting in poor communication between professional groups, who work independently [24]. The findings of the current study suggest that it is collaboration between leads from different specialties that provides a platform on which to co-design hip fracture pathway guidelines, protocols and service improvement initiatives. Furthermore this pathway needs to be embedded into working practice through training and induction. The importance of involving all members of the MDT in the development and use of pathway documentation are detailed in BOA guidelines [9] and previous research [23]. The current study advocates that understanding the pathway documentation should be a core part of junior doctor and nursing inductions.

Literature highlights the necessity of good communication across the MDT in order to coordinate care and implement the pathway [5]. The importance of frequent contact in fostering collaboration between clinical teams has been highlighted as a means to improve service of quality [11]. The current study emphasizes the variety and combination of communication modes which facilitate coordinated MDT care, integrating the hip fracture pathway at all stages (See Table 4). Face to face ward-based meetings and ‘huddles’ can be augmented by information sharing methods, such as live bed status metrics bed management tools, live trauma boards and WhatsApp. Findings support the importance of staff having access to computers, hand-held devices or mobile phones.

**Table 4:**
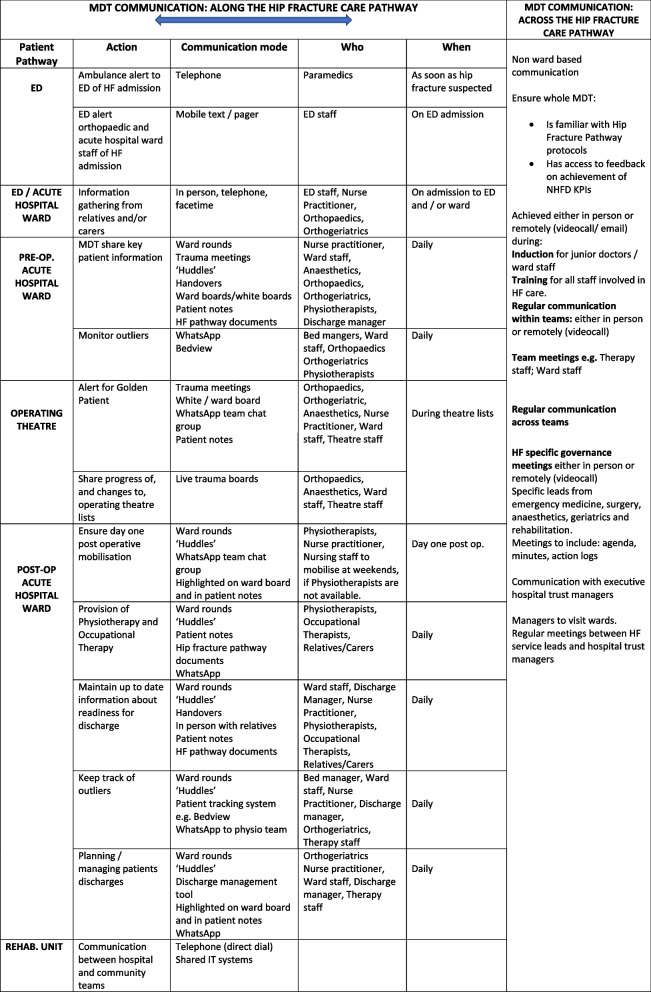
Communication strategies along and across the hip fracture pathway

The necessity of an appropriately staffed orthogeriatric service is paramount [5]. Orthogeriatricians provide pre and post operative medical management and are skilled in the optimisation of co-morbidities in frail older people. They provide continuity of care throughout the patient’s hospital admission, liaising with other specialties as well as family and carers. Methods to recruit and retain orthogeriatricians are suggested, including advertising, job planning and sufficient timetabling for hip fracture patient care. Evidence suggests that some aspects of the Orthogeriatrician role are increasingly implemented by specialist hip fracture nurses [25]. In the current study, specialist hip fracture nurses played a central role, linking members of the MDT and to collating NHFD performance data.. The findings emphasise the importance of resourcing services with these key staff members, who are specifically trained to coordinate care, and support the high nursing and therapy needs of this patient group.

Specific initiatives to overcome barriers to care delivery include strategies to improve the efficiency of trauma lists, such as flexible use of elective theatres and routine identification of ‘golden patients’. Consistent use of fascia-iliaca nerve blocks is recommended by the National Institute for Health and Care Excellence [26]. Our findings highlight strategies to ensure their consistent use in the ED. This is timely, given recent evidence indicating nerve block use pre-surgery is associated with reduced length of hospital stay [27]. The value of coordinated, multidisciplinary discharge planning, daily discharge reviews and shared information systems between hospital and community settings is also emphasised.

The qualitative findings reported here will complement the quantitative results of the NAME study [15]; identifying factors that contribute to unwarranted variation in patient outcomes. A Hip Fracture Implementation Toolkit will be co-produced in collaboration with the Royal Osteoporosis Society and other stakeholders. Once developed it is intended that these tools will be freely available to all NHS organisations, to assist services in overcoming organisational barriers when commissioning and implementing high-quality fracture services [15].

### Strengths and limitations

Qualitative interviews elicited in-depth experiences of healthcare professionals at four diverse UK hospitals. As some professionals could only comment on specific aspects of the hip fracture pathway, criterion sampling ensured adequate coverage. It is possible that because different numbers of participants were recruited at each hospital, some could have been over-represented in the analysis. To mitigate this, data from each hospital were analysed separately and then compared. Analysis of 23 BOA reports added a wider reach to the study and their structure complimented the in-depth interviews, as each source provided unique information. It is a methodological strength that both data sources were double coded by three experienced qualitative researchers. The PPI group feedback specifically highlighhted the potential for suggested service improvements to improve the experiences of patients and families. We chose not to apply a theoretical framework, such as those derived from the field of implementation science [28] to this work. Instead, we opted to structure the barriers and facilitators we identified into domains along and across the care pathway. This was a pragmatic approach that enabled us to develop recommendations for care delivery at each stage of the pathway and will help to facilitate the use of findings into clinical practice. Further findings relating to multi-disciplinary working within hip fracture care that builds on existing theories and frameworks will be presented in another article. Discussion of findings with the research stakeholder and Patient and Public Involvement (PPI) groups provided assurance that findings were sufficiently transferable to other settings.

## Conclusion

This study examined organisational processes that help or hinder the implementation of key components of hip fracture services. It echoes evidence of the multiple organisational factors affecting patient outcomes [27] and complements existing guidelines, providing information about strategies to achieve effective hip fracture care in daily clinical practice. Based on these findings, a set of recommendations have been generated on how to deliver effective hip fracture care (Table 5), which may help hospitals to design and implement effective services to improve patient care.

**Table 5:** Recommendations

ALONG THE HIP FRACTURE CARE PATHWAY		
Domain	National target/ standard	Recommendations
Emergency Department Admission	Admission to an acute hip fracture ward within 4 h of presentation (BOA)	1. Promptly notify orthopaedic, orthogeriatric and nursing staff of new hip fracture patients in the ED 2. Plan for specialist nurse(s) to monitor ED presentations, coordinate admissions and rapid ward transfers 3. Conduct training to routinely deliver fascia iliaca nerve blocks in ED, by ED staff, with equipment reliably accessible in ED
Pre-operative Care		1. Provide care for hip fracture patients in designated hip fracture wards 2. Deliver care through specialist trained teams
	Perform surgery within 36 h (BPT) Perform surgery on the day of, or the day after, admission (NICE)	1. Ensure coordinated MDT decision-making when planning surgery 2. Agree that patients should not be cancelled by any one single member of the MDT, but only after consultation with at least one other senior member of the MDT 3. Ensure anaesthetists review patients early before an operation to maximise optimisation of patients 4. Trauma lists should: ➢ Be published early (ideally the night before) ➢ Start with a ‘golden patient’ who has been prepared the day before ➢ Include an extra list on a Friday ➢ Have access to elective theatre space when needed ➢ Be monitored, with progress and changes communicated promptly to the MDT via agreed routine information systems, such as a live electronic trauma feeds
Post-operative care	All patients presenting with a fragility fracture should be managed on a hip fracture ward with routine access to acute orthogeriatric medical support from the time of admission (NICE; BPT)	1. Provide timetabled orthogeriatrican input for all hip fracture patients 2. Train specialist hip fracture nurses (ANPs) to upskill nursing staff *e.g*., patient mobilisation 3. Provide specific hip fracture training for ward-based junior doctors, including explaining targets for care 4. Hip fracture nurses and orthogeriatricians should provide support to junior doctors from medicine and surgery 5. Physiotherapists and occupational therapists should undertake joint patient assessments and maintain shared plans for patient rehabilitation and discharge 6. Agree discharge destination early and review plans daily with MDT members 7. Employ discharge coordinators to work 7 days a week 8. Provide training so that a range of health professionals can undertake falls assessments *e.g*., ACPs, physiotherapists
ACROSS THE HIP FRACTURE CARE PATHWAY		
Domain	Target	Recommendations
Communication & Coordination	Multidisciplinary management (NICE) Adults with hip fracture are cared for within a Hip Fracture Programme at every stage of the care pathway	1. Co-design hip fracture pathway documents collaboratively between clinical leads from orthopaedics, orthogeriatrics, anaesthetics, physiotherapy, occupational therapy and nursing teams 2. Explain hip fracture pathway documents at induction and training of junior doctors and nurses 3. Ensure documents are used and completed daily by MDT members 4. Provide shared, or adjacent office space, for MDT members 5. Organise daily face-to-face meetings or ‘huddles’, ward-based meetings and daily board rounds between MDT members 6. Invest in efficient joined-up IT systems for information sharing between the MDT 7. Ensure reliable electronic communication systems are available to monitor theatre activity and manage outliers, *e.g*., ‘bedview’
Service improvement and clinical governance:	Clinical and service governance which is responsible for all stages of the pathway of care and rehabilitation, including those delivered in the community. (NICE)	1. Allocate time for hip fracture clinical leads to develop robust clinical governance processes 2. Name a hospital-level executive lead, who reports to Trust board, to promote hip fracture care delivery as a Trust priority 3. Hold regular, minuted, hip fracture governance meetings to drive service improvement, with attendance from orthopaedic, orthogeriatric, anaesthetic, therapy and nursing teams 4. Allocate time for (3) within professional job plans and provide access to management training and managerial support to drive service improvement 5. Nominate specific personnel to consistently enter national hip fracture audit data, with time for this role specified in their job plan 6. Routinely monitor service performance against national targets/standards and feedback results regularly to the MDT 7. Trigger automatic investigations when targets/standards are not met and/or if mortality increases 8. Conduct regular mortality reviews, with documentation of learning points and routine feedback to the MDT

## Data Availability

Anonymised interview data can be accessed via the University of Bristol Research Data Repository. Access to the data will be made available to researchers for ethically approved research projects, from a six year embargo period, which ends on 1st April 2029. Data can be accessed on the understanding that confidentiality will be maintained and after a Data Access Agreement has been signed by an institutional signatory. No authentic request for access will be refused. Data can be accessed via the University of Bristol Data Repository using the following link: https://data.bris.ac.uk/data/dataset/3f9j9z626s7f32a3r5j1wd7zt7. To request access to this data after the embargo period, please contact Professor Rachael Gooberman-Hill: R.Gooberman-Hill@bristol.ac.uk. Access to the British Orthopaedic Association peer reviewed reports will be closed since reports are the intellectual property of the British Orthopaedic Association and were made available on a prior exclusive agreement.
