# Correction: Complex organisational factors influence multidisciplinary care for patients with hip fractures: a qualitative study of barriers and facilitators to service delivery

**DOI:** 10.1186/s12891-024-07839-7

**Published:** 2024-09-13

**Authors:** F Fox, S Drew, CL Gregson, R Patel, TJS Chesser, A Johansen, MK Javaid, XL Griffin, R Gooberman‑Hill

**Affiliations:** 1 Bristol Medical School, University of Bristol, Bristol, UK; 2 Department of Trauma and Orthopaedics, North Bristol NHS Trust, Bristol, UK; 3 University Hospital of Wales and School of Medicine, Cardiff University, Cardiff, UK; 4 Nuffield Department of Orthopaedics, Rheumatology and Musculoskeletal Sciences, University of Oxford, Oxford, UK; 5 Bone and Joint Health, Barts and The London School of Medicine and Dentistry, Queen Mary University of London, London, UK; 6 Barts Health NHS Trust, London, UK; 7 National Institute for Health Research Bristol Biomedical Research Centre, University Hospitals Bristol and Weston NHS Foundation Trust and University of Bristol, Bristol, UK

**Correction** **:** **BMC Musculoskelet Disord 24, 128 (2023)**

**https://doi.org/10.1186/s12891-023-06164-9**

Following publication of the original article [1], the authors corrected two errors in Table 1. The table shows 4 orthogeriatricians and 0 physiotherapists at the site with pseudonym 'Springhill'; these have been corrected to 3 orthogeriatricians and 1 physiotherapist.

The original article [1] has been corrected.
